# Comparative Transcriptome Analysis Reveals that a Ubiquitin-Mediated Proteolysis Pathway Is Important for Primary and Secondary Hair Follicle Development in Cashmere Goats

**DOI:** 10.1371/journal.pone.0156124

**Published:** 2016-10-03

**Authors:** Xiao-yang Ji, Jian-xun Wang, Bin Liu, Zhu-qing Zheng, Shao-yin Fu, Getinet Mekuriaw Tarekegn, Xue Bai, Yong-sheng Bai, Heng Li, Wen-guang Zhang

**Affiliations:** 1 College of Animal Science, Inner Mongolia Agricultural University, Hohhot, 010018, China; 2 Institute of ATCG, Nei Mongol Bio-Information, Hohhot, 010020, China; 3 State Key Laboratory of Genetic Resources and Evolution, Kunming Institute of Zoology, Chinese Academy of Sciences, Kunming, 650223, China; 4 Animal Research institution of Animal Science Academy of XinJiang Uygur Autonomous Region, Urumqi, 830001, China; 5 Inner Mongolia Academy of Agricultural & Animal Husbandry Science, Hohhot, 010031, China; 6 Department of Biology, The Center for Genomic Advocacy, Indiana State University, Terre Haute, Indiana, 47809, United States of America; 7 Department of Microbial, Cellular and molecular biology, Addis Ababa University, Addis Ababa, Ethiopia; 8 Department of Animal production and Technology, Biotechnology Research Institute, Bahir Dar University, Addis Ababa, Ethiopia; 9 College of Life Sciences Inner Mongolia Agricultural University, Hohhot, 010018, China; China Agricultural University, CHINA

## Abstract

**Background:**

The fleece of cashmere goats contains two distinct populations of fibers, a short and fine non-medullated insulating cashmere fiber and a long and coarse medullated guard hair. The former is produced by secondary follicles (SFs) and the later by primary follicles (PFs). Evidence suggests that the induction of PFs and SFs may require different signaling pathways. The regulation of BMP2/4 signaling by noggin and Edar signaling via Downless genes are essential for the induction of SFs and PFs, respectively. However, these differently expressed genes of the signaling pathway cannot directly distinguish between the PFs and SFs.

**Results:**

In this study, we selected RNA samples from 11 PFs and 7 SFs that included 145,525 exons. The pathway analysis of 4512 differentially expressed exons revealed that the most statistically significant metabolic pathway was related to the ubiquitin–mediated proteolysis pathway (UMPP) (P<3.32x 10^−7^). In addition, the 51 exons of the UMPP that were differentially expressed between the different types of hair follicle (HFs) were compared by cluster analysis. This resulted in the PFs and SFs being divided into two classes. The expression level of two selected exons was analyzed by qRT-PCR, and the results indicated that the expression patterns were consistent with the deep sequencing results obtained by RNA-Seq.

**Conclusions:**

Based on the comparative transcriptome analysis of 18 HFs from cashmere goats, a large number of differentially expressed exons were identified using a high-throughput sequencing approach. This study suggests that UMPP activation is a prominent signaling pathway for distinguishing the PFs and SFs of cashmere goats. It is also a meaningful contribution to the theoretical basis of the biological study of the HFs of cashmere goats and other mammals.

## Background

Cashmere goats have a double coat consisting of the over hairs, or guard hairs, produced by primary hair follicles (PFs) and the under hairs, or down hairs, produced by the secondary hair follicles (SFs)[1,2]. PFs and SFs are formed during the fetal stages of growth. From 55–60 days of age, PFs form in two steps (central PFs form first and then lateral PFs form to create a trio group). SFs form from 90–100 days of age. Thereafter, derived SFs are formed from branches of the PFs and from epidermal SFs as the keratinized epidermis (from 115–120 days of gestation) is not able to produce new epidermal the hair follicles (HFs, the PFs and SFs collectively referred to as HFs)[3]. The growth cycle of SFs is similar to that of PFs, both of them pass through three stages of growth in its complete growth cycle[4]. These stages areactive growth (anagen), regression (catagen) and quiescence (telogen). But at the end of telogen, when molting occurs and both the PFs and SFs shed their fibers, a sparse coat of mainly guard hairs is maintained while the cashmere fibers are almost completely detached [1,5]. These characteristics make the cashmere goat an ideal model system for studies of the morphology and development of HFs.

However, how differentially expressed genes can be used to distinguish the PFs and SFs is poorly understood. There is only minimal information in the scientific literature on gene expression in goat HFs, and efforts need to be made to define the gene expression patterns associated with fiber growth. Meanwhile, the mammalian HF may provide valuable information for understanding processes of genetic and molecular regulation. Several studies suggest that different signal regulation mechanisms may affect the development of different types of HFs. BMP signaling by noggin is required for the induction of SFs, but the induction of the PFs has been shown to not be affected by BMP, which is consistent with the previous report that showed that the tumor necrosis factor receptor homologue Edar was required for the induction of this HF subtype[6–8]. In recent years, additional research has further demonstrated that ubiquitination is associated with the development of HFs. Ubiquitination causes myriad functional changes because of post-translational modifications to proteins, such as phosphorylation[9]. This suggests that regulation of protein-protein interactions can be facilitated by polyubiquitination, which ultimately causes changes in the activity of transcription factors and the subcellular localization of transcriptional cofactors[10–12]. Meanwhile, new data point to protein polyubiquitination as a mechanism for regulating the stability and interaction of the key signaling components that control HF development and regeneration[12].

In the current study, because of the “noise” of the transcriptome, we have compared the differential expression of genes analysis by RNA-Seq, but the genes were not significantly enriched in any signaling pathway could distinguish PFs from SFs. Finally, by taking advantage of an exon-based transcriptome analysis, we found that characteristics of the UMPP can directly distinguish PFs and SFs. Exon-based transcriptome analysis suggests that significantly different exons are produced from the large number of total expressed exons. The exon-transcriptome analysis indicated that a functional mammalian transcriptome could be detected and characterized by examination of the exon expression across normal tissues because most known genes consist of exons and are expressed at different levels across different tissues and developmental states. In addition, exon analysis offers improved sensitivity and may allow for more accurate quantitative measurements of the level of gene expression[13].

Transcriptome sequencing of HFs was carried out to identify various types of HFs at the transcription level. Exons and exon-networks that were thought to be involved in the divergence of HFs were examined. Moreover, functional divergence of the genes and enriched expressions were examined to determine the genes involved in the development of HF types.

## Results

### Transcriptome profiling of primary hair follicles and secondary hair follicles

In the quantification of the exon expression patterns, large variations (ranging from 7.0% and 8.4%) existed between individual HFs (PFs and SFs collectively referred to as HFs) in the percentage of coexpressed exons. To identify exon expression differences between primary hair follicles (PFs) and secondary hair follicles (SFs), we selected RNA samples from 11 PFs and 7SFs encompassing 145,525 exons. We analyzed the differential exon expression profiles and identified 4512 exons with significantly different expression between the two types of HFs(P<0.01).

### Functional classification of exon-significant differences

To determine the functions of the exons differentially expressed between the PFs and SFs, we identified 3972 genes from the 4512 exons with significantly different expression patterns and performed aBlast2GO analysis via a search of the NR database. The genes by Gene Ontology (GO) terms according to group across categories of biological processes, cellular compartments, and molecular functions[14].

**Fig 1:**
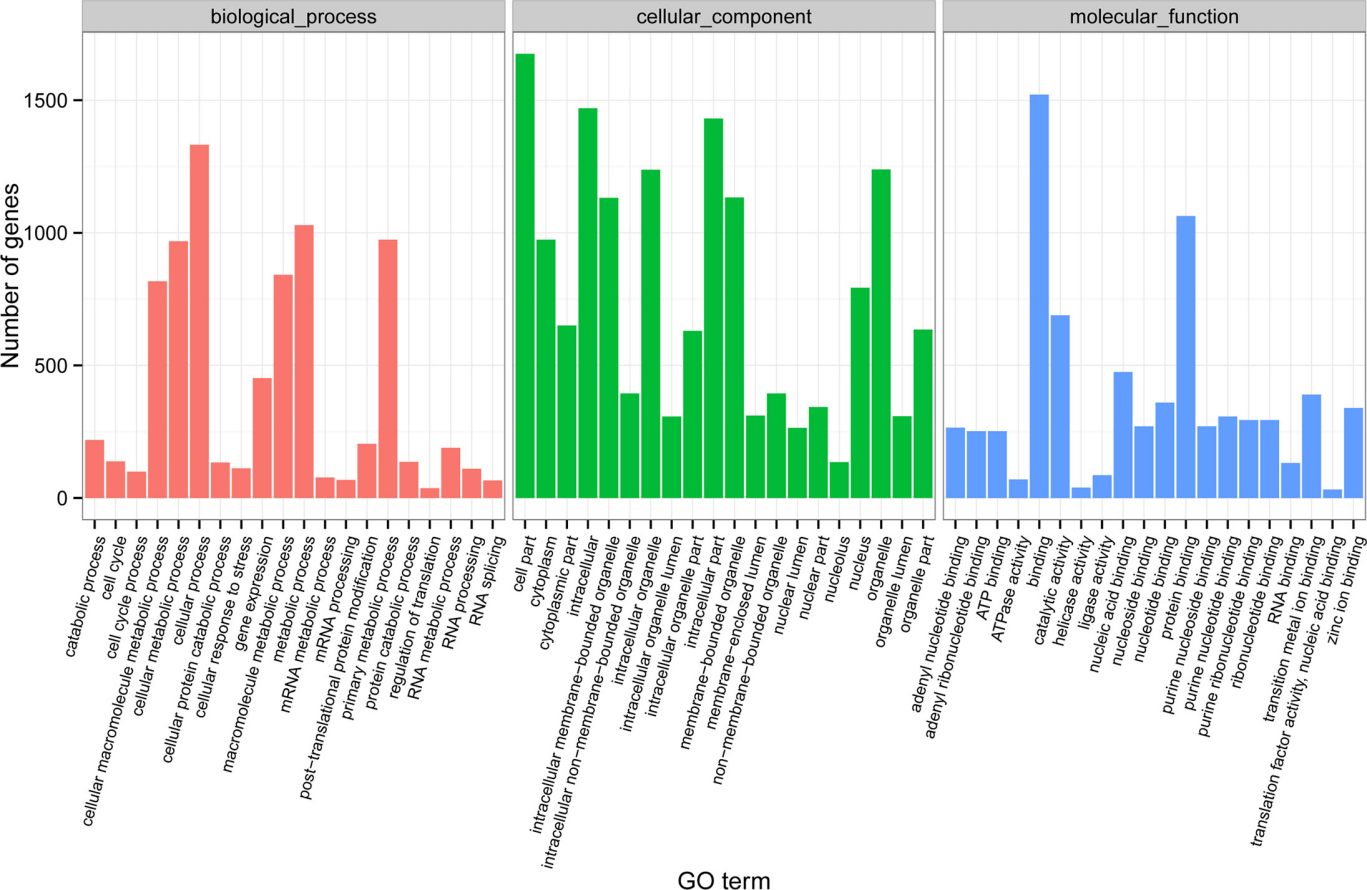
Histogram of the GO classifications of gene-significant differences. Results of significantly different genesare summarized for three main GO categories: biological process, cellular component and molecular function.

Using the GO analysis, the differentially expressed genes were determined to be enriched globally, regardless of species affiliation. For example, the development of different types of HFs was shown to involve genes in the enriched categories described below based on the dataset comparison (Fig 1). The products of most genes were distributed broadly throughout the nucleus, cytoplasm, and organelles. Among the functional group of biological processes, the PF- and SF-related genes that Mediate Function were significantly enriched in categories related to primary metabolic process, regulation of translation, RNA metabolic process, protein catabolic process.

**Fig 2:**
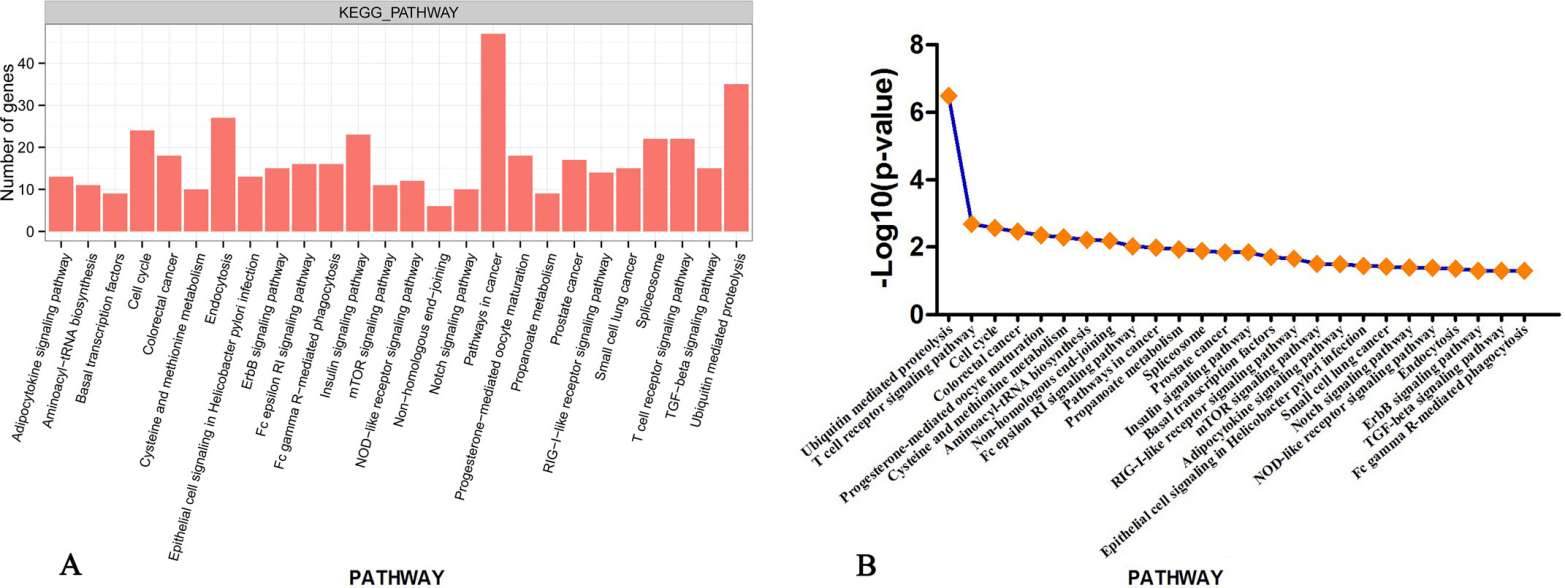
Summary of gene-significant differences in KEGG pathways. (A)Distribution of the KEGG pathways of gene-significant differences is shown as a bar chart. The number of gene hits is shown along the Y-axis while the different KEGG pathways are shown along the X-axis. P<0.05 was used as the thresholds in selecting significant KEGG pathways.The 26 significantly different KEGG terms are shown. (B) P-value for KEGG pathways.

To examine the signaling pathways of the genes involved in the different interactomes identified between the PFs and SFs, we analyzed our data using the KEGG pathway database and the DAVID bioinformatics tool [14], and a total of 3972 genes with differential expression patterns were placed into the following KEGG pathway categories (Fig 2A): Notch signaling pathway, TGF-beta signaling pathway and cell cycle processes. This suggests that there are considerable differences between the physiological processes in different types of HFs in cashmere goats. Meanwhile, the *SKP1* gene of the UMPP was related to the functions of the TGF-beta pathway. Fig 2B shows, in the line graph, the statistically significant differences (p<0.05 and–log10 (fold change)) between 26 KEGG pathways. Notably, the KEGG pathway analysis of the genes with the greatest difference in expression showed that they were significantly enriched with regard to the UMPP (P<3.32x 10^−7^).

### Cluster correlation analysis of the UMPP exons

**Fig 3:**
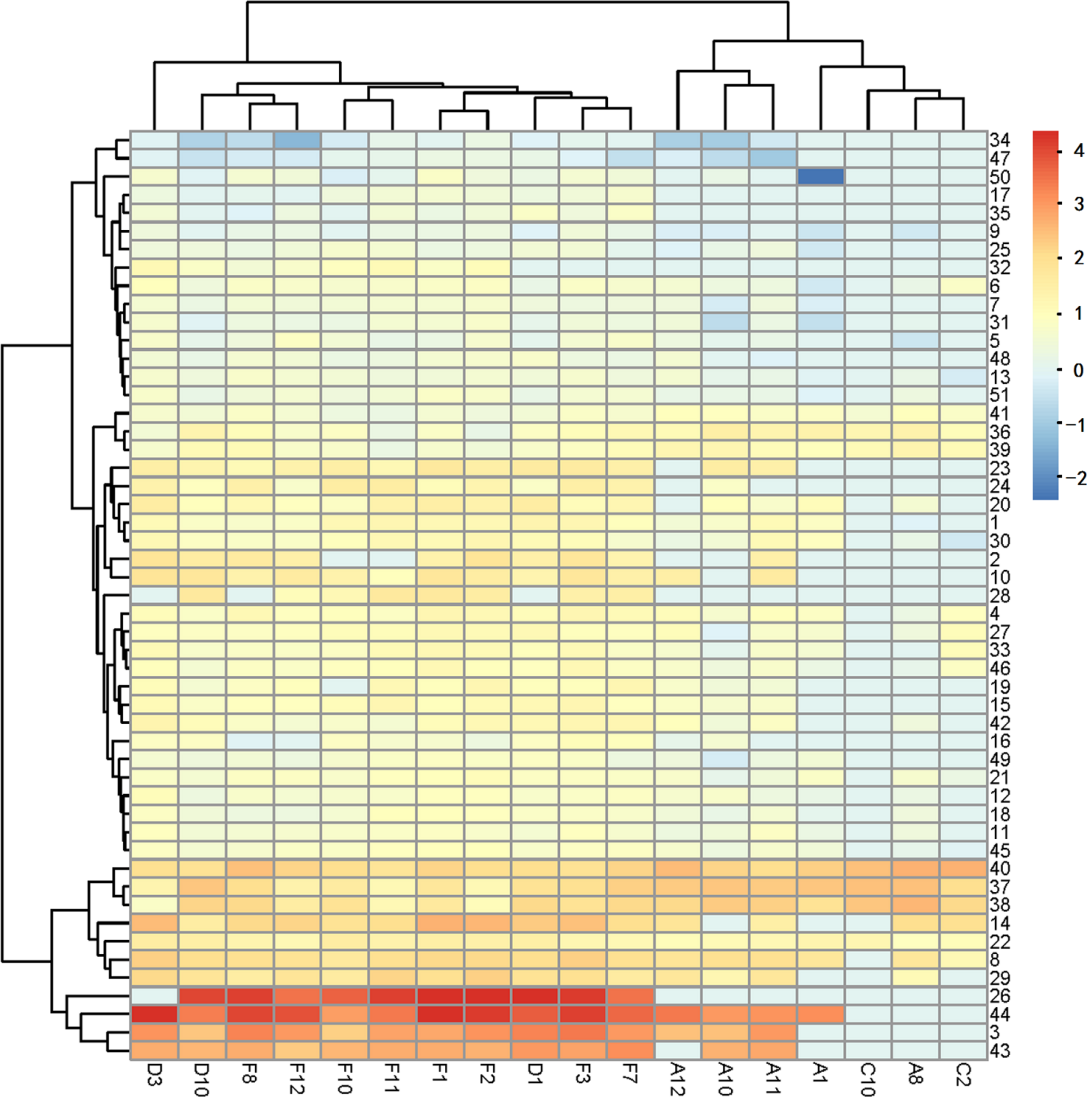
Pairwise correlation between all samples. Based on the differential exon expression, the heatmap exhibits a clustering of 18 transcriptome samples (HF type*exon). It represents differentially expressed exons between distinct types of HFs. Color map is used to visualize the differences in expression, ranging from blue (normalized expression of −2) to red (normalized expression value of 4).

To measure the correlations between the differential expression patterns of exons in the samples, we identified 51 exons in the UMPP showing significantly different expression patterns between the 11 PFs and 7 SFs (S1 Table). The cluster analysis included 44 exons that were present at higher levels and 7 exons that were present at lower levels in PFs. The cluster correlation analysis of PF- and SF-divergent exons is shown in Fig 3, which indicates that they are clearly separated based on the UMPP-related exons and are clustered into two classes. This indicates that the UMPP might be valuable as a discriminator between the PFs and SFs. On the other hand, we found only 7 exons that were expressed in all 18 samples, which were also related to the *UBE2O* gene. This indicates that exon expression changes are steady in HFs, which will be the focus of further analyses.

### Exon co-expression network analysis in UMPP exons

**Fig 4:**
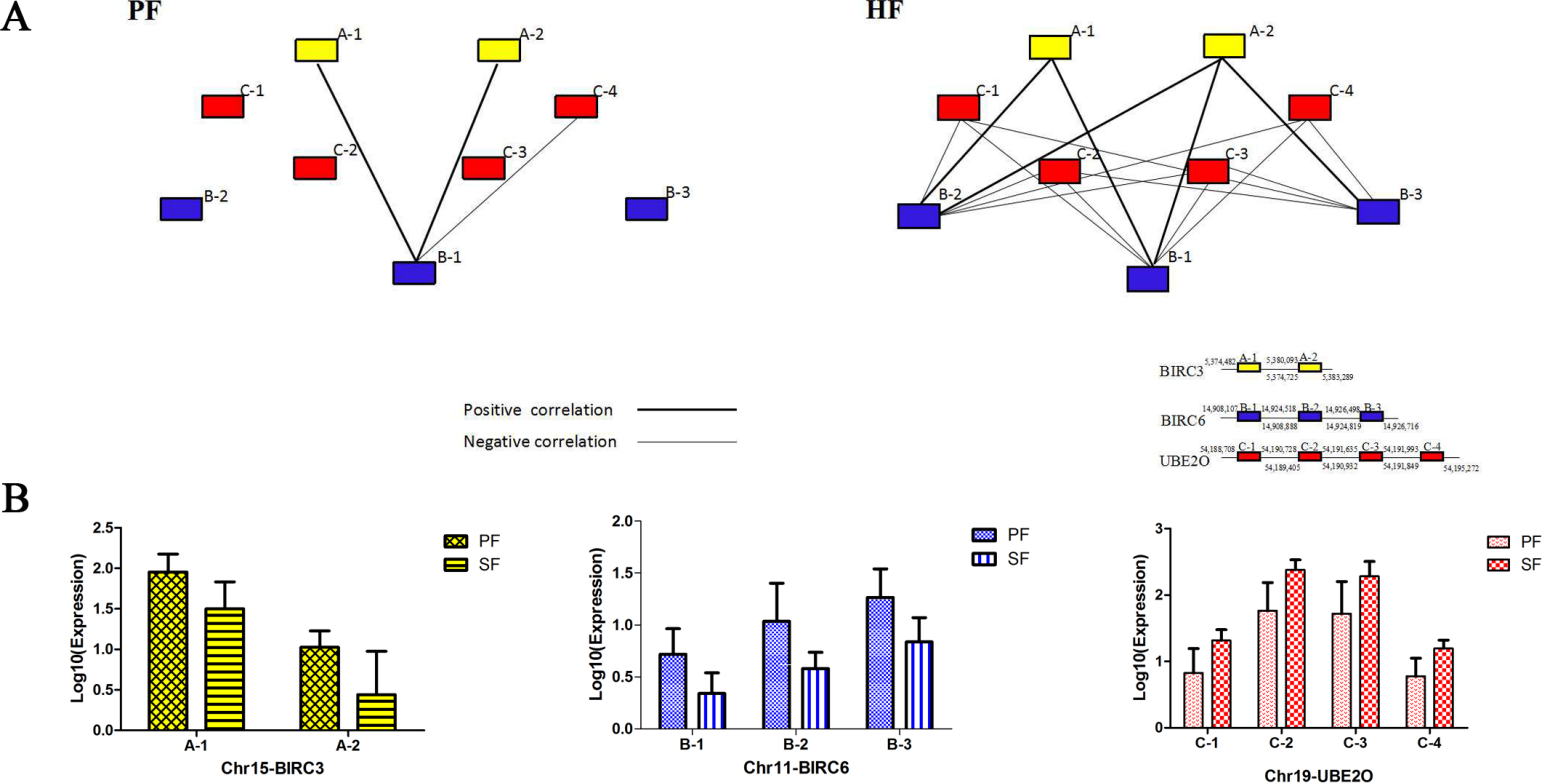
The co-expression network of differentially expressed exons. (A) Nodes represent the exons, the edges between them represent the exon-exon interaction, and the thick and thin lines indicate positive and negative correlations, respectively. The exons considered for the network assembly are highlighted with red, yellow and blue boxes. Red nodes represent *UBE2O*; yellow nodes indicate *BIRC3* and the blue nodes represent *BIRC6*. The positions of exons have been indicated in annotations.The figures represent the starting position above the line and the ending position below. (B)The exonexpression of *BIRC3*, *BIRC6* and *UBE2O* in PFs and SFs.The *BIRC3*, *BIRC6* and *UBE2O* are labeled in yellow, blue and red, respectively. The expression of exons is shown along the Y-axis while the different positionson the chromosome are shown along the X-axis.

Here, we used a UMPP exon co-expression network analysis in a first attempt to identify the PF- and SF-associated co-expression modules and their key constituents. As the SF sample was limited, it could not be used to determine the exon expression correlations based on the SF co-expression network, we therefore estimated the exon correlations in the SFs based on the HF and PF networks. We used the correlation coefficients of the expression of 51 non-redundant exons to calculate the pairwise correlations between exons. To avoid the effect of confounding factors on the computed correlations, we used only the correlation coefficients that showed significant changes based on t-tests (P<0.05) and found three genes with large differences in their range of node degree variation, which were further analyzed for co-expression. We examined the associations between the 9 exons of these 3 genes (*UBE2O*, *BIRC3*, *BIRC6*) in relation to their node degrees, which showed substantial differences between the two exon networks (Fig 4) and the functional associations of the nine points predicted to be involved in the development of HFs. Combined with the correlation coefficients for exon expression, we found that *UBE2O*was negatively correlated with *BIRC6* and that *BIRC3* and *BIRC6* had a positive correlation. The exons in *UBE2O* compared with those of *BIRC6* showed significantly different correlations in both HFs and PFs. The comparison showed differences in the relationships between the 9 exons, which indicated markedly different degrees of exon co-expression and different patterns in HFs and PFs. Then, the co-expression networks were used to screen the core genes that may be involved in distinguishing the PFs and SFs.

### Validation of the differences in expression of the selected genes identified by RNA-Seq in the RNA samples isolated from the PFs and SFs using qRT-PCR

**Fig 5:**
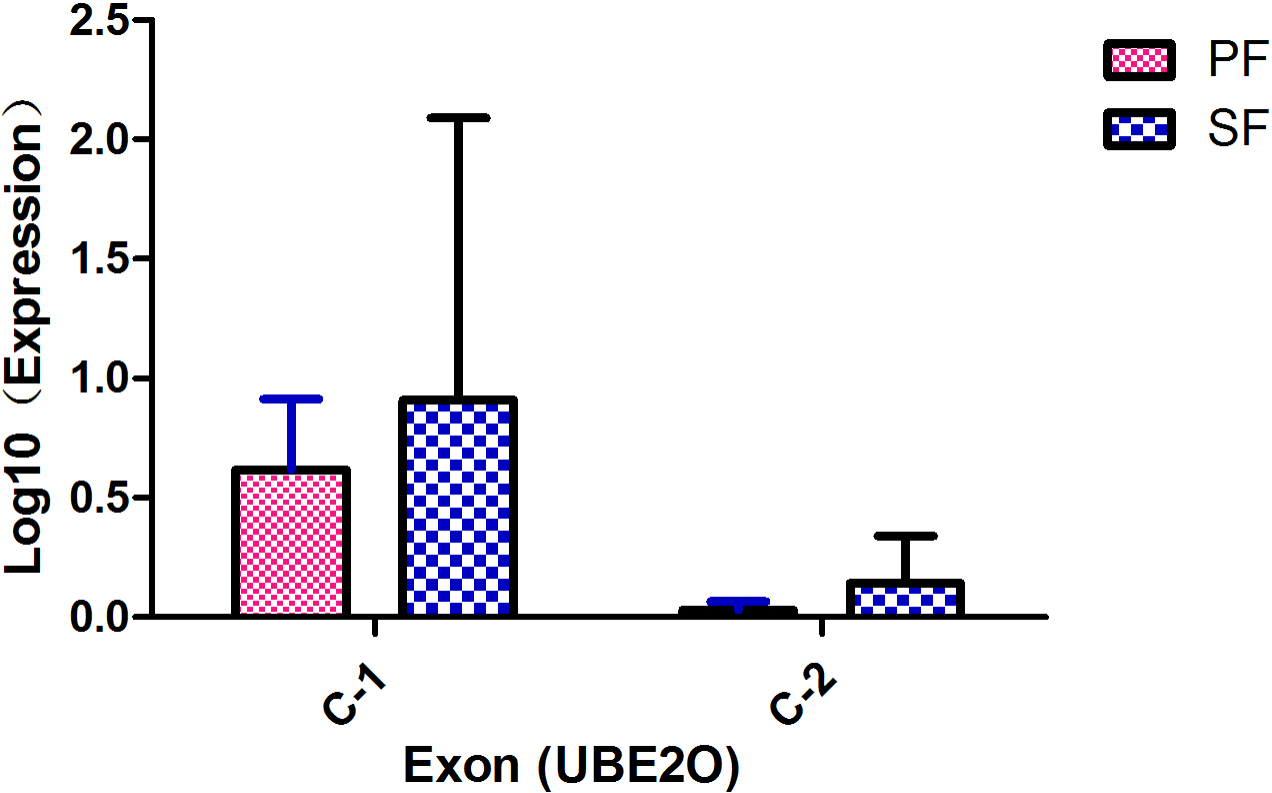
qRT-PCR validation of exon expression among the different types of HF samples.

As another approach to determine RNA-Seq expression patterns and levels of expression, we performed quantitative RT-PCR for the different types of HFs from cashmere goats using specific primers (Fig 5). In this experiment, we eventually selected two exons of the *UBE2O* gene using the scarce samples of SFs for comparison with the results of the analysis above. The qRT-PCR result demonstrate that there are significant differences in the expression patterns between the PFs and SFs. The C-1 exon and C-2 exon of *UBE2O* (P<0.05) showed higher levels of expression in SFs than in PFs. The comparative analysis of the qRT-PCR and RNA sequencing results of these two exons shows that the qRT-PCR results were consistent with the exons analysis results. This indicates that the observed differences in exon expression between PFs and SFs based on transcript abundance were well supported.

## Discussion

The Illumina high throughput sequencing platform is widely used for transcriptome analysis based on the sequencing-by-synthesis (SBS) technique [15]. This is because of the high throughput, accuracy, repeatability and low signal-to-noise ratio of this sequencing platform[16–18]. Here, we provided a comprehensive insight on the PF and SF transcriptomes of cashmere goat fibers using Illumina deep sequencing. Recently some cases have been reported that Zhu et al. showed the differentially expressed genes between primary and secondary hair follicle-derived dermal papilla cells cultured in vitro involved in vascularization, ECM-receptor interaction and Wnt/β-catenin/Lef1 signaling pathways[19]. However, based on the different exon expression patterns of 18 RNA-Seq libraries between primary and secondary hair follicles which plucked from the vivo environment, we identified the ubiquitin–mediated proteolysis pathway (UMPP).The main function of the UMPP is to degrade proteins. This degradation is performed in two main steps. The first step involves a large number of ubiquitin molecules being attached to the substrate. The second step involves the 26S proteasome and the recycling of ubiquitin, which degrades the tagged protein through the activity of ubiquitin C-terminal hydrolases[20–22]. Our studies demonstrates that the UMPP could be useful for the further characterization of novel molecules associated with the regulation of HFs. It has long been recognized that HF formation and maintenance depend on reciprocal interactions between specialized dermal cells and epidermal stem cells[23,24]. The fleece consists of a number of different types of HFs characterized by differences in their morphology and the time-course of their induction during embryogenesis[25–27]. The interaction of numerous growth stimulating and inhibiting factors controls the growth of HFs. Some of the signaling pathways that facilitate the growth of the HFs are the Wnt, Shh, Notch, TGF-β, Edar and Bmp pathways[24,28,29]. Studies have indicated that the Wnt, Shh and NF-kB/EDAR pathways are crucial for the growth and maintenance of HFs[30–33]. Increasing evidence suggests that the induction of PFs and SFs may require different signaling pathways. Headon and Overbeek (1999) stated that the induction of PFs depends on signaling through the Tnf receptor homologue Edar. The regulation of BMP signaling by noggin is essential for the induction of SFs, as well as for the advanced stages of development in PFs[7,8].

It has previously been shown that the ubiquitination of proteinsis crucial in regulating the timing, duration, and location of pathway signaling. In the Wnt signaling pathway, the best-known example of polyubiquitination acting as a signaling mechanism is in the control of β-catenin protein levels. Polyubiquitination also plays a central role in the regulation of the Shh/Gli and NF-KB/TRAF signaling pathways during hair morphogenesis. The polyubiquitination signaling pathway regulates NF-κB activity at almost all steps of the signal transduction process. These results suggest that it may also play an important role in the signaling pathways involved with different types of HFs in cashmere goats. Recent data suggest that ubiquitination plays a much broader role in regulating protein function, such as the ubiquitination of the hair differentiation regulator Notch and the degradation of the Notch intracellular domain[34]. Similarly, ubiquitin-dependent degradation plays critical roles in the regulation of TGF-β[35,36] and the E3 ubiquitin ligases also regulate the TGF-β family signaling[37].

Although the function of the UMPP in the different types of HFs remains to be fully defined, we provide evidence in the present study that *UBE2O* plays an important role in the control of development-associated exon expression in the PFs and SFs. An analysis of the co-expression networks indicates that the fifteenth exon (C-2 exon) belongs to the gene *UBE2O*. Based on the large differences in node degree, our data suggest that this exon is closely associated with the regulation SF development. In addition, the qRT-PCR results demonstrated that *UBE2O* was over-expressed in SFs compared to the expression in PFs, which is consistent with the sequencing analysis results. One intriguing possibility is that the *UBE2O* gene may participate in the different signaling pathways of HF development and may play a different role in the regulation of each type of HF. We suggest that *UBE2O* functions as a positive regulator in BMP7 signaling and that BMP signaling promotes the induction of SFs[7,38]. Moreover, *UBE2O* acts as a novel negative regulator of TRAF6-dependent NF-KB signaling in multiple cell types and additional biochemical studies have confirmed that interactions with EDAR result in NF-KB activation[6,39–41]. Interestingly, Eda and Edar in combination with Downless or Tabby mutants result in defects in HF induction, leading to a lack of development of PFs. These genes in the UMPP may lead to the different patterns of signal regulations that subsequently affect the development and morphogenesis of PFs and SFs. However, this hypothesis requires further research.

The accuracy of the identification of the genes corresponding to most enriched pathway and GO terms were confirmed by the Cluster exon-expression network and qRT-PCR analysis, and51 of the selected exons showed significant differences. Consequently, these results strongly support the use of our RNA-Seq data as a reliable resource for further analysis of the differential patterns of gene expressions. Taken together, our data demonstrate a previously unrecognized role of the UMPP in the complex regulation of the differential exon expression that distinguishes the PFs and SFs. Although many aspects of the UMPP-dependent-effects on HFs remain to be clarified, these data may help in further exploring the role of the UMPP in the development of distinct types of HFs.

## Conclusions

The comparative transcriptome analysis of 18 HFs from cashmere goats revealed 4512 exons that are differentially expressed between PFs and SFs. An integrated analysis of differential gene expression, functional classification and cluster correlation, and co-expression networks suggest that UMPP activation is a prominent signaling pathway for distinguishing the PFs and SFs of cashmere goats. This study also provides a meaningful contribution to the theoretical basis of the biological study of the HFs of cashmere goats and other mammals.

## Methods

### Experimental animals and sample collection

The experimental procedure was approved by the Animal Care and Use Committee of Inner Mongolia Agricultural University, China and was performed in accordance with the animal welfare and ethics guidelines. Experimental cashmere goats were obtained from Nei Mongol. All cashmere goats were raised using feeding practices based on standard practices for the care of cashmere goats. Protocols used in this experiment were consistent with those approved by the Institutional Animal Care and Use Committee of Inner Mongolia Agricultural University.

As there is no obvious cycle in the primary hair follicles (PFs), we collected according to the growth cycle of the secondary hair follicles (SFs). HFs (PFs and SFs collectively referred to as HFs) were collected from anagen to telogen of the cashmere goat hair cycle. The PFs and SFs were collected and selected from 4 cashmere goats in each period, respectively. In total we collected 12 PFs and 12 SFs over entire year. After iodine disinfection and alcohol deiodination, we carefully separated the primary hair follicle and secondary hair follicle, then the primary and secondary follicles were identified through the associated structures. The hair follicles were quickly plucked from the dermis by hand[42]. The PFs and SFs plucked from the side of the torso of female Nei Mongol cashmere goats were immediately frozen without any chemical solutions in liquid nitrogen for storage and transport until used for RNA isolation[2].

We collected and selected the 11 PFs and 7 SFs from 4 cashmere goats due to the scarcity of the samples and mRNA extraction difficulties. The PFs samples were collected at three hair follicle developmental stages (anagen, catagen and telogen) and the SFs samples were collected specifically at anagen and catagen. Both the PFs and SFs were mixed respectively and then compared within groups, and cannot be compared correspondingly.

### RNA extraction, sequence library construction, and illumina sequencing

RNA from each PF or SF sample group was extracted with TRIzol reagent (TaKaRa, Dalian city, China) according to the manufacturer's instructions, and purity and degradation were checked on 1% agarose gels. DNA was removed from the RNA extracts by incubation with RNase-free DNase for 30 min at 37°C.

A cDNA library was constructed and oligo (dT) magnetic beads (Illumina) were employed to isolate Illumina sequencing poly(A) mRNA isolated from total RNA. The purified mRNA was broken into short fragments using a fragmentation buffer. Random hexamer primers and reverse transcriptase (Illumina) were used to perform first-strand cDNA synthesis using the short fragments as templates. Second-strand cDNA was synthesized using RNase H (Illumina), DNA polymerase I (Illumina), dNTPs and buffer. These cDNA fragments were subjected to an end-repair process and ligation to adapters. These products were purified and enriched with PCR to create the final cDNA library.

### Sequence preprocessing and functional annotation

To obtain clean data, all sequenced raw data were processed, which included removing the adapter and filtering out the low quality reads and the proportion with N more than 10% using in-house perl script. Then, all clean data were mapped to the goat genome using TopHat2[43] with no discordant and mixed parameters. Unique mapped reads were retained to estimate the exon abundances in a downstream analysis. Reference-guided transcriptome assembly, which compensates for incompletely assembled transcripts, was performed by Cufflinks[43] with a bias correction for each sample, and then, the data were merged into a single unified transcript catalog using Cuffmerge. Isoforms with an abundance below 0.1 were discarded to remove the low-quality transcripts. Differentially expressed exons were estimated by Cuffdiff2[43] (exon center) using RPKM (reads per kilobase per million mapped reads) based on P-value<0.01 and FDR<0.05.

### Enrichment analysis

To explore the functional annotation and pathway enrichment of the significantly different genes in the interactomes and compare them between the PFs and SFs, we used the online analysis tool DAVID (the Database for Annotation, Visualization and Integrated Discovery, Version6.7)[44] to determine the enriched Gene Ontology (GO) terms (P-value<0.0001) and the Kyoto Encyclopedia of Genes and Genomes (KEGG) (P-value<0.05), in which we focused on the KEGG feature.

### Differentially expressed exon analysis

A rigorous algorithm was used to identify exons that were shown by a t-test to be significantly differentially expressed[45]. We used a P-value corresponding to a differential exon expression test at statistically significant levels. A P-value<0.01 was used as the threshold to identify differently expressed exons.

Cluster correlation analysis was used to demonstrate differentially expressed exons between the different types of HFs. The procedure was as follows: Fold changes (log 10 Ratio) were estimated according to the normalized exon expression level in each sample. The exon co-expression correlation was calculated and identified for differentially expressed exons using Pearson's correlation with a cutoff P-value (P<0.01) and custom R scripts were written to identify the degree of exon nodes for further analysis. A pairwise t-test[45] was employed to compare differences in the degrees of the exons expressed in PFs and SFs.

### Quantitative real-time PCR (RT-PCR) validation

We selected another 23 PFs and 9 SFs from the RNA-Seq analysis of 4 cashmere goats, they were collected over the entire year, snap frozen in liquid nitrogen, and stored at −80°C for the subsequent qRT-PCR analysis. Total RNA was extracted from PFs and SFs using TRIzol (TaKaRa) following the manufacturer's protocols (Thermo Scientific NanoDrop 2000). Approximately 0.5 μg of total RNA was used as a template to synthesize the first-strand cDNA with a Primer Script RT reagent Kit (TaKaRa) following the manufacturer's protocols (S2 Table). The resultant cDNA was diluted to 0.1 μg/μl for further analysis via qRT-PCR (Bio-Rad) using SYBR Green Realtime PCR Master Mix (TaKaRa). *GAPDH*[46] was chosen as internal reference gene to eliminate sample-to-sample variations. The relative quantitative method 2−ΔΔCt was to compare the expression differences in processed and untreated samples. As this study did not involved in the untreated samples, we just used the application of 2−ΔCt method add standard curve to estimate the exon expression. Where ΔCt = (Ct_1_-Ct_2_) and in presented for both the exon of UBE2O and GAPDH. The presence of significant differences in expression between PFs and SFs were determined by GLM analysis using SAS software 9.0[45].

## Supporting Information

S1 Table The expression of 51 exons in 18 samples ofPFs and SFs. (XLS)

S2 Table Primers for real time qRT-PCR. (DOC)

## Abbreviations

UMPP: ubiquitin-mediated proteolysis pathway
HFs: hair follicles
PFs: primary hair follicles
SFs: secondary hair follicles
RNA-Seq: RNA-sequencing
GO: Gene ontology
KEGG: Kyoto Encyclopedia of Genes and Genomes
qRT-PCR: quantitative real-time polymerase chain reaction
SBS: sequencing-by-synthesis
RPKM: reads per kb per million reads
SAS: statistical analysis system
NR: non-redundant
FDR: false discovery rate
DAVID: Database for Annotation, Visualization and Integrated Discovery
cDNA: complementary DNA
GLM: generalized linear models
