# Supplementary material for: Comparative Transcriptome Analysis Reveals that a Ubiquitin-Mediated Proteolysis Pathway Is Important for Primary and Secondary Hair Follicle Development in Cashmere Goats
Source: PLoS One. 2016 Oct 3;11(10):e0156124. doi: 10.1371/journal.pone.0156124 (PMC5047472; doi:10.1371/journal.pone.0156124)
Supplement: S2 Table — (DOC) [file pone.0156124.s002.doc]

**Table S3**, Primers for real time qRT-PCR.

| **Gene** | **Primer Number** | **Primer Sequence (5’-3’)** | **Annealing Temperature(℃)** | **Product size(bp)** |
| --- | --- | --- | --- | --- |
| UBE2O  (C-1) | chr19_54189405-F | GACTACGACTCGGTGGAAGG | 64℃ | 118 |
|  | chr19_54189405-R | CCTCTATCTTGGGGTGCTCAT | 64℃ |  |
| UBE2O  (C-2) | chr19_54190932-F | GGACCTCTTCTCGGCCTTGA | 64℃ | 189 |
|  | chr19_54190932-R | CCCAGGAGGCTGACGCACA | 64℃ |  |
| GAPDH | GAPDH - F | TCCACGGCACAGTCAAGG | 58℃ | 112 |
|  | GAPDH - R | TCAGCACCAGCATCACCC | 58℃ |  |
